# Development of a novel short 12-meric papiliocin-derived peptide that is effective against Gram-negative sepsis

**DOI:** 10.1038/s41598-019-40577-8

**Published:** 2019-03-07

**Authors:** Jieun Kim, Binu Jacob, Mihee Jang, Chulhee Kwak, Yeongjoon Lee, Kkabi Son, Sujin Lee, In Duk Jung, Myeong Seon Jeong, Seung-Hae Kwon, Yangmee Kim

**Affiliations:** 1 Department of Bioscience and Biotechnology, Konkuk University, Seoul, 05029 South Korea; 2 Department of Immunology, Lab of Dendritic Cell Differentiation and Regulation, School of Medicine, Konkuk University, Chungju, 27478 South Korea; 3 Chuncheon Center, Korea Basic Science Institute, Chuncheon, 24341 South Korea

## Abstract

The development of novel peptide antibiotics with potent activity against multidrug-resistant Gram-negative bacteria and anti-septic activity is urgently needed. In this study, we designed short, 12-meric antimicrobial peptides by substituting amino acids from the N-terminal 12 residues of the papiliocin (Pap12-1) peptide to alter cationicity and amphipathicity and improve antibacterial activity and bacterial membrane interactions. Pap12-6, with an amphipathic α-helical structure and Trp^12^ at the C-terminus, showed broad-spectrum antibacterial activity, especially against multidrug-resistant Gram-negative bacteria. Dye leakage, membrane depolarization, and electron microscopy data proved that Pap12-6 kills bacteria by permeabilizing the bacterial membrane. Additionally, Pap12-6 significantly reduced the secretion of NO, TNF-α, and IL-6 and secreted alkaline phosphatase reporter gene activity confirmed that Pap12-6 shows anti-inflammatory activity via a TLR4-mediated NF-κB signaling pathway. In a mouse sepsis model, Pap12-6 significantly improved survival, reduced bacterial growth in organs, and reduced LPS and inflammatory cytokine levels in the serum and organs. Pap12-6 showed minimal cytotoxicity towards mammalian cells and controlled liver and kidney damage, proving its high bacterial selectivity. Our results suggest that Pap12-6 is a promising peptide antibiotic for the therapeutic treatment of Gram-negative sepsis via dual bactericidal and immunomodulatory effects on the host.

## Introduction

A major global public health threat, costing thousands of lives and substantial economic damage, is the emergence of antibiotic resistance in pathogenic bacteria^1–3^. New antibiotics for Gram-negative bacterial infections, in particular, are urgently needed^4,5^. The outer membrane of Gram-negative bacteria consists of lipopolysaccharides (LPS) and acts as a permeability barrier, making the development of antibiotics challenging^5^. Gram-negative bacterial infection leads to the release of LPS, which are recognised by toll-like receptor 4 (TLR4) on macrophages, subsequently triggering the overexpression of cytokines and an uncontrolled inflammatory response, leading to sepsis^6–8^. Gram-negative sepsis results from a harmful host response to infection, in which bacteria and LPS released from bacteria activate immune cells, such as monocytes and macrophages^9^. It is extremely difficult to develop a specific therapeutic agent against bacterial cells during sepsis. Accordingly, there is an immediate and urgent need for the development of alternative antibiotics^10^.

Antimicrobial peptides (AMPs) are small peptides that are part of the innate immune system of various organisms^10–12^. In addition to antimicrobial activity, they have immunomodulatory effects^13,14^. Many AMPs are active against multidrug-resistant (MDR) pathogenic bacteria and mainly target the bacterial cell membrane, making them a promising new class of therapeutic agents^15–17^. AMPs can modulate the host immune response in multiple ways, including the recruitment of immune cells to the site of infection. The immunomodulatory properties of AMPs can be exploited to treat inflammation and sepsis^10,18,19^. Several AMPs are known to exert antiendotoxic activities and suppress LPS-induced proinflammatory cytokine production^19,20^. Colistin suppresses proinflammatory cytokines in the LPS-challenged condition but shows high cytotoxicity^19^. Certain LPS antagonists derived from lactoferrin, cathelicidins, granulysins, and β-boomerang peptides can directly interact with LPS and neutralise its endotoxic action^11,20^.

Cecropins are a major class of AMPs found in insects^21^. Papiliocin, a cecropin found in swallowtail butterfly larvae^22^. Our previous study revealed that it has a helix-hinge-helix structure and is active against MDR Gram-negative bacteria, with low toxicity to mammalian cells^23^. Trp^2^ and Phe^5^ contribute to its antibacterial and anti-inflammatory activity^23,24^. Papiliocin permeabilizes the bacterial cell membrane, different from the mode of action of conventional antibiotic molecules. These abilities make papiliocin a highly attractive therapeutic candidate; however, its use may be limited due to its length of 37 residues, which can make production costs high. Short peptides with antimicrobial activity can reduce the cost of production and cause low immunogenicity making them ideal candidates for therapeutic applications. Therefore, in this study, we aimed to design and synthesise potent 12-meric peptides derived from the 12 amino acids in the N-terminus of papiliocin (Pap12-1). We evaluated their antibacterial and anti-inflammatory activities *in vitro* and *in vivo* and investigated the mechanism underlying their antibacterial activities. In particular, we found that Pap12-6 can significantly inhibit bacterial growth and suppress inflammatory cytokine production *in vitro* and *in vivo*. These short dodecapeptides are promising novel peptide antibiotic candidates for the treatment of sepsis caused by Gram-negative bacterial infection.

## Results

### Peptide design

**Figure 1:**
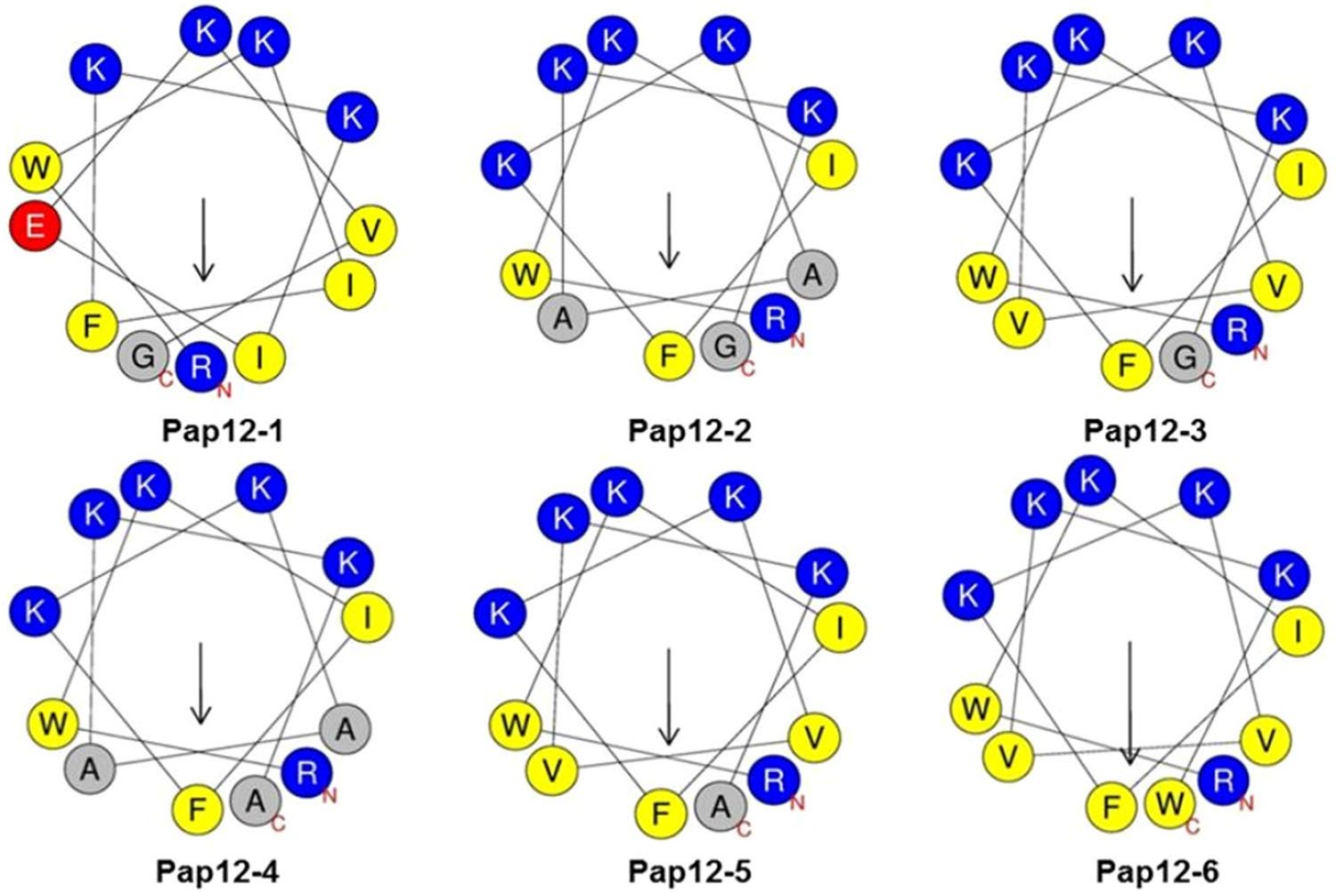
Helical wheel diagram of Pap12 peptides generated using HeliQuest^47,48^. Positively charged residues are presented in blue and negatively charged residues are shown in red. Hydrophobic residues are shown in yellow below the wheel and Gly and Ala are shown in grey. The arrows represent the helical hydrophobic moment.

To develop short novel peptide antibiotics showing potent antibacterial activity and anti-sepsis activity, we designed 12-meric analogues from the N-terminal 12 residues of papiliocin (Pap12-1), as listed in Table 1^23–25^. Previous studies have reported 12-meric peptides with potent antibacterial activities derived from long α-helical AMPs, such as MSI-78 and LL-37^26,27^. Additionally, 12-meric peptides with antiendotoxic activity have been designed with boomerang-like β-strand conformations^28^. The dodecamer represents an optimal length for antibacterial activities, while still allowing peptides to retain amphipathic secondary structures. The N-terminal 12 residues of papiliocin were chosen, since this was the minimum length to retain meaningful antibacterial activity (MIC of 16 μM) against *Escherichia coli* and because it containsTrp^2^ and Phe^5^, which have been identified as key residues for papiliocin antimicrobial activity^23–25^. In this study, Val^11^ of Pap12-1 at the boundary of the hydrophobic phase was substituted with Lys to increase the net positive charge as well as amphipathicity in all analogues (Fig. 1). The cationicity and amphipathicity of AMPs play important roles in mediating the initial interaction with the negatively charged bacterial cell membrane^29^. To evaluate the effect of hydrophobicity on peptide activity, Ala or Val were substituted for Ile^8^ and Glu^9^ in the hydrophobic phase, resulting in increase of cationicity to +6 in all analogues. Pap12-2 was obtained from Pap12-1 by substituting Ala in the 8^th^ and 9^th^ positions, while Pap12-3 was designed by replacing the 8^th^ and 9^th^ amino acids with Val, resulting in a higher hydrophobicity compared to that for Ala. It has been reported that valine substitution can improve antimicrobial activity without increasing cytotoxicity^30^. Furthermore, Gly^12^ at the 12^th^ position was replaced with Ala or Trp to improve membrane interactions. Thus, Pap12-4 was derived from Pap12-2, in which Gly^12^ was replaced with Ala, thereby improving amphipathicity. Pap12-5 was obtained from Pap12-3 by replacing Gly^12^ with Ala. Finally, Pap12-6 was designed by substituting Gly^12^ with Trp to increase hydrophobicity and improve membrane interactions. It has been reported that Trp residues confer more potent antimicrobial activity than phenylalanine or tyrosine^31^. Further, the indole ring in Trp interacts with the interfacial region of a membrane, allowing AMPs to partition well into the bilayer interface^32,33^. While Pap12-1 has four lysine residues on the hydrophilic face, all analogues with Lys at the 11^th^ position had an extended angle at the hydrophilic face, with increased cationicity, as shown in Fig. 1. The hydrophobic phase of the peptides was also modified by the substitution of Gly^12^ with Ala or Trp residues in analogues, resulting in higher amphipathicity, which can improve antibacterial activity via better interactions with the membrane. Large mean amphipathic moments of peptides, listed in Table 1, indicate that the helix is amphipathic perpendicular to the helix axis. Pap12-6 had the greatest mean amphipathic moment among the six peptides; its positively charged amino acid residues were located on the upper polar surface and hydrophobic amino acid residues were on the other side of the helical axis. Theoretical and measured molecular weights of the peptides are listed in Table 1, proving that all peptides were synthesised successfully.

**Table 1:** Peptide designs and physicochemical properties.

Peptide Name	Sequence	Charge	µH^a^	Calculated MW	Measured MW^b^
Pap12-1	RWKIFKKIEKVG-NH2	+4	0.486	1531.9	1530.8
Pap12-2	RWKIFKKAAKKG-NH2	+6	0.459	1460.8	1459.8
Pap12-3	RWKIFKKVVKKG-NH2	+6	0.555	1516.9	1515.8
Pap12-4	RWKIFKKAAKKA-NH2	+6	0.483	1474.8	1473.8
Pap12-5	RWKIFKKVVKKA-NH2	+6	0.580	1530.9	1529.9
Pap12-6	RWKIFKKVVKKW-NH2	+6	0.736	1646.1	1645.0
Melittin	GIGAVLKVLTTGLPALISWIKRKRQQ-NH2	+5	0.394	2847.49	2846.49

^a^μH represents the mean amphipathic moment or hydrophobic moment and was calculated using the HeliQuest site at: http://heliquest.ipmc.cnrs.fr/cgi-bin/ComputParams.py.

^b^Molecular weight (MW) measured by mass spectroscopy.

### Antimicrobial activity

The antimicrobial activities of Pap12 peptides were tested against four standard Gram-negative bacteria (*E. coli, Pseudomonas aeruginosa, Acinetobacter baumannii, and Salmonella typhimurium*), three standard Gram-positive bacteria (*Staphylococcus aureus, Bacillus subtilis*, and *Staphylococcus epidermidis*), and five MDR bacteria (MDREC CCARM 1229, MDRPA CCARM 2002, MDRAB CCARM 12035, MDRST CCARM 8007, and MRSA CCARM 3126) using the microbroth dilution method. The relative antibacterial activities were compared based on geometric means of MICs (GM). All designed peptides with a cationicity of at least +6 showed higher antibacterial activity compared with that of Pap12-1 (Table 2). The replacement of C-terminal Gly^12^ with Trp resulted in a dramatic improvement in antibacterial activity and GMs of Pap12-6 against all bacteria were more than five times stronger than those of Pap12-1. GMs of peptides against Gram-negative bacteria tended to increase in the following order: Pap12-6 (6) > Pap12-5 (14) > Pap12-3 (16) > Pap12-2 (18) > Pap12-4 (22) > Pap12-1 (35). The high antibacterial activity of Pap12-3, 5, and 6 showed good correlation with their high amphipathic moments. GMs of peptides against Gram-positive bacteria tended to increase in the following order: Pap12-6 (7) > Pap12-5 (14) > Pap12-3 (14) > Pap12-2 (20) > Pap12-4 (22) > Pap12-1 (52). All analogues with Val^8^ and Val^9^ in the hydrophobic phase (Pap12-3, 5, and 6) showed significantly higher antibacterial activities than those of other peptides. Despite the short length of dodecapeptide, Pap12-6 showed high potency against Gram-negative bacteria; its MIC values were comparable to those of melittin, the well-known bee venom peptide.

**Table 2:** Antibacterial activities of Pap12 peptides

Microorganisms	Minimal inhibitory concentration (MIC) (μM)						
	Pap12-1	Pap12-2	Pap12-3	Pap12-4	Pap12-5	Pap12-6	Melittin
**Gram-negative**							
*E. coli*	16	8	8	8	8	4	4
*P. aeruginosa*	8	8	8	8	8	4	8
*A. baumannii*	64	32	16	32	8	4	8
*S. typhimurium*	64	16	16	32	32	8	4
*MDREC 1229*	64	32	16	32	16	8	8
*MDRPA 2002*	16	16	32	32	16	8	4
*MDRAB 12035*	16	16	16	16	8	4	4
*MDRST 8007*	32	16	16	16	16	8	8
GM^a^	35	18	16	22	14	6	6
HC_10_	200	200	200	200	200	100	0.8
Therapeutic index^b^	5.7	11.1	12.5	9.1	14.3	16.7	0.1
**Gram-positive**							
*S. aureus*	16	16	8	8	8	4	4
*B. subtilis*	64	16	16	16	16	8	2
*S. epidermidis*	64	32	16	32	16	8	4
*MRSA 3126*	64	16	16	32	16	8	2
GM^a^	52	20	14	22	14	7	3
HC_10_	200	200	200	200	200	100	0.8
Therapeutic index^b^	3.8	10.0	14.3	9.1	14.3	14.3	0.3

^a^GM is the geometric mean of MICs against all bacteria. ^b^Therapeutic index = HC_10_/MIC, where HC_10_ is the peptide concentration resulting in 10% hemolysis of fresh human erythrocytes. When no detectable haemolytic activity was observed at the highest concentration tested, two times the maximum concentration was used to calculate the therapeutic index. Larger values indicate greater cell selectivity.

### Cytotoxicity against mammalian cells

**Figure 2:**
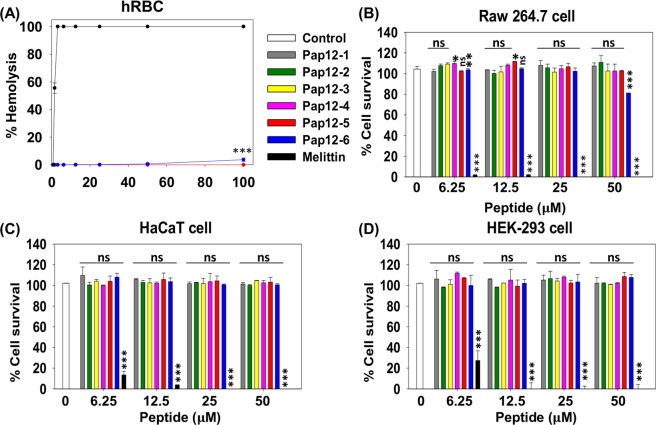
Toxicities of Pap12 peptides measured as (**A**) hemolysis in human red blood cells and (**B**) cytotoxicity against mouse macrophage RAW 264.7 cells, (**C**) human keratinocyte HaCaT cells, and (**D**) HEK-293 human embryonic kidney epithelial (HEK-293) cells. All measurements were obtained in triplicate. Error bars represent means ± SEM. (*p < 0.05; **p < 0.01; ***p < 0.001; n.s. represents no significance).

The toxicities of peptides against hRBCs are shown in Fig. 2A. All peptides, except Pap12-6, showed no hemolysishemolysis at up to 100 µM, while melittin was highly toxic against hRBCs. Pap12-6 did not show hemolysis at up to 50 µM, and only 3% hemolysis was observed at 100 µM. The therapeutic index (TI) was calculated (Table 2) using HC_10_, the peptide concentration required to induce 10% hemolysis. HC_10_ values of 200 µM were used for all peptides that did not show hemolysis at 100 µM, while that of Pap12-6 was 100 µM. All analogue peptides showed higher TIs than that of the parent peptide Pap12-1. TIs of peptides against Gram-negative bacteria tended to decrease in the following order: Pap12-6 (16.7) > Pap12-5 (14.3) > Pap12-3 (12.5) > Pap12-2 (11.1) > Pap12-4 (9.1) > Pap12-1 (5.7). Pap12-6 had the highest TI and high bacterial cell selectivity.

The toxicities of peptides against mammalian cells were checked by measuring the survival rate of mouse macrophage RAW 264.7 cells, human keratinocyte HaCaT cells, and HEK-293 human embryonic kidney epithelial (HEK-293) cells after treatment with different concentrations of peptides (Fig. 2B–D). Using RAW 264.7 cells, Pap12-6 peptides resulted in 100% cell survival at up to 25 µM and 80% survival at 50 µM, while all other peptides resulted in 100% survival rates against RAW 264.7 cells at 50 µM. All peptides, including Pap12-6, resulted in 100% cell survival at their MICs and showed no cytotoxicity against HaCaT and HEK-293 cells, even at 50 µM, implying that they are not cytotoxic against human cells. Melittin, on the other hand, displayed cytotoxicity at very low concentrations. Since melittin has higher hydrophobicity and a lower amphiphatic moment (µH) than those of Pap12 peptides, as listed in Table 1, its toxicity is much higher than those of Pap12 peptides. These results indicate the peptides are potent antibiotics with high antimicrobial activity and low toxicity.

### Secondary structures of peptides, as determined by CD spectroscopy

**Figure 3:**
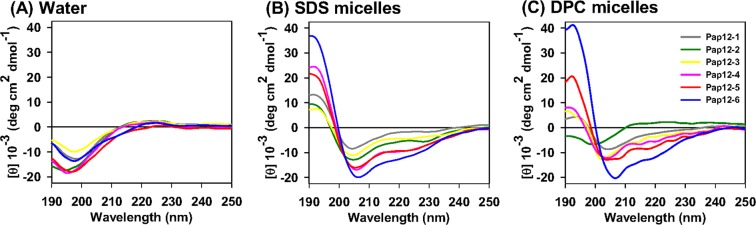
CD spectra for 50 μM Pap12 peptides in (**A**) aqueous solution, (**B**) 100 mM SDS micelles, and (**C**) 50 mM DPC micelles.

Pap12 peptides showed random coil structures in aqueous solution and adopted an α-helical structure in the presence of micelles. In particular, Pap12-5 and 6, with high amphipathic moments, showed α-helical structures in membrane-mimetic environments, such as SDS micelles and DPC micelles, as evidenced by characteristic double minima in the spectra (Fig. 3). Secondary structure formation in the membrane-mimetic environment shows the ability of the peptides to interact with the membrane. Pap12-6 with high antibacterial activity had high α-helicity, indicating that the amphipathic α-helical structure plays important roles in bacterial cell membrane permeabilization. These results were confirmed by molecular modelling to predict the α-helical structures of the peptides using PEP-FOLD, a *de novo* peptide structure prediction server, as shown in Fig. S3^34–36^. Pap12-6 which has the highest mean amphipathic moment shows amphiphatic α-helical structure.

### Peptide-induced dye leakage

**Figure 4:**
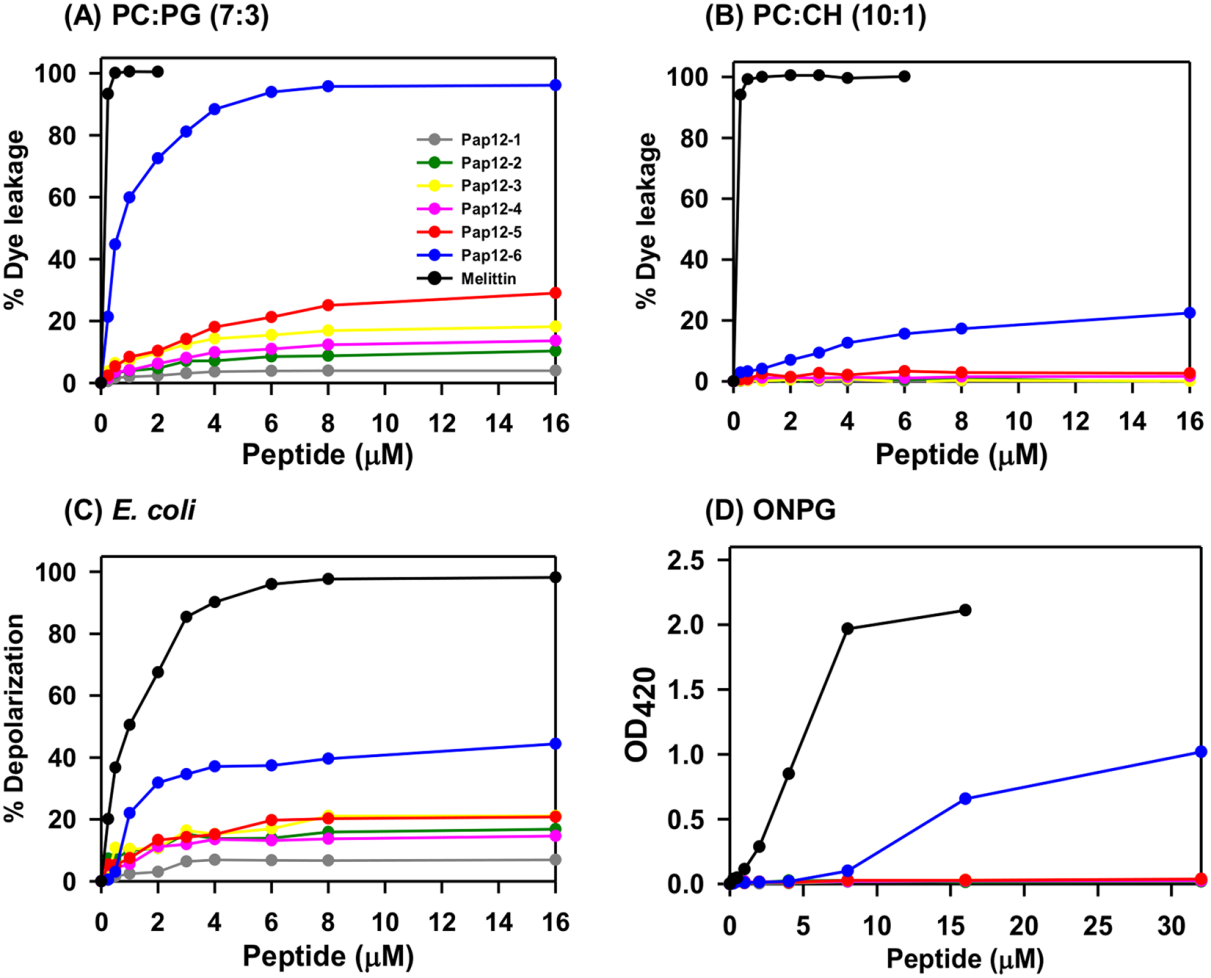
Peptide-induced dye leakage from different LUVs with entrapped calcein and membrane depolarization measurements. Peptide-induced dye leakage from (**A**) bacterial membrane mimicking PC:PG = 7:3 LUVs and from (**B**) mammalian membrane mimicking PC:CH = 10:1 LUVs. Peptide-induced membrane depolarization measured with the DiSC_3_(5) dye against (**C**) intact *E. coli*, and (**D**) inner membrane permeability assessed by ONPG assays on *E. coli* ML35p cells.

To determine the mechanism of antibacterial action, we evaluated the ability of peptides to permeabilize bacterial membranes by measuring the peptide-induced leakage of the fluorescent dye calcein from the model membranes (Fig. 4A,B). All peptides, except Pap12-6, did not induce substantial calcein release and showed poor membrane permeabilization in PC/CH (10:1, w/w) LUVs as well as PC/PG (7:3, w/w) LUVs. Pap12-6, however, showed 100% leakage from bacterial cell-mimicking LUVs and 22% leakage at 16 µM from mammalian-mimicking PC/CH LUVs, implying that Pap12-6 can target bacterial cell membranes with high selectivity. The control peptide melittin permeabilized all LUVs, implying that melittin is not selective for bacterial cells.

### Membrane depolarization

The membrane depolarization capacity is a measure of bactericidal efficiency. The depolarization abilities of the peptides were measured by calculating the amount of membrane potential sensitive dye released against intact *E. coli*. Among all Pap12 peptides, Pap12-6 (16 µM) showed the highest depolarization capacity against intact *E. coli*, with 44% depolarization (Fig. 4C). An ONPG assay was employed to measure the peptide-induced inner membrane permeability of *E. coli* ML35p strains (Fig. 4D). Chromophore yields from cytoplasmic ONPG were measured as an indicator of the ability of the peptide to permeabilize the inner membrane of *E. coli*. Bacteria were treated with various concentrations of peptides (ranging from 0.25 to 32 µM) and colour formation was observed. The Pap12-6 peptide resulted in the greatest colour development, implying that it permeabilizes the inner membrane most efficiently among all peptides. These results suggested that Pap12-6 targets the bacterial cell membrane, while other peptides may have different modes of action, since they showed poor membrane depolarization abilities. High membrane depolarization activity of Pap12-6 can probably be attributed to its high amphipathicity and the effective hydrophobic partitioning of Trp^12^ in the bacterial cell membrane.

### Visualization of *E. coli* membrane disruption by Pap12-6 using SEM micrographs

**Figure 5:**
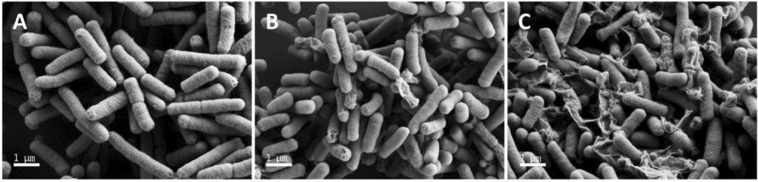
Scanning electron micrographs of *E. coli* treated with Pap12-6. FE-SEM micrographs of *E. coli*: (**A**) control, without peptide. (**B**) Pap12-6 at 1× MIC. (**C**) Pap12-6 at 2× MIC. The scale bar represents 1.0 μm. Magnification was 30000×.

Dye leakage data as well as membrane depolarization data suggested that Pap12-6 exerts antibacterial activity by permeabilization of the bacterial cell membrane. To confirm the antibacterial mechanism of Pap12-6, we visualised *E. coli* membrane damage by field emission scanning electron microscopy (FE-SEM). Control cells exhibited smooth surfaces (Fig. 5A). Figure 5B,C show the SEM images of *E. coli* after treatment with the 1× MIC or 2× MIC of Pap12-6 for 2 h, respectively. The membrane surfaces of *E. coli* became rough and wrinkled upon 1× MIC Pap12-6 treatment (Fig. 5B) and were further wrinkled and corrugated upon 2× MIC treatment, as shown in Fig. 5C, compared with the control (Fig. 5A).

### Inhibition of NO production and inflammatory cytokine production in LPS-stimulated RAW 264.7 cells

**Figure 6:**
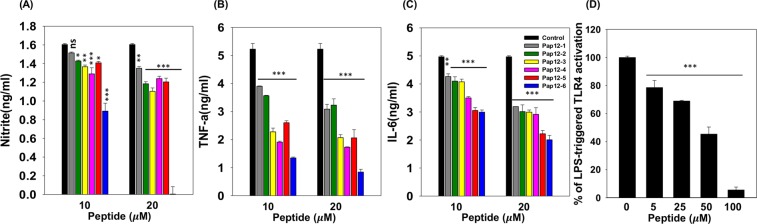
Anti-inflammatory activities of Pap12 peptides. (**A**) Inhibition of nitrite production by peptides (10 μM and 20 μM) in LPS (50 ng/mL)-stimulated RAW 264.7 cells. (**B**) Levels of mTNF-α and (**C**) mIL-6 in the supernatant of LPS (20 ng/mL)-stimulated RAW 264.7 cells exposed to Pap12 peptides for 16 h. (**D**) Dose-dependent reduction of SEAP activity showing the suppression of activation of NF-κB in 20 ng/mL LPS stimulated HEK-Blule™ hTLR4 cells. The cells were pretreated with Pap12-6 for 1 h and then treated with LPS for 14 h. SEAP activity of culture supernatants with different concentration of Pap12-6 (0, 5, 10, 25, 50, 100 μM) were tested and were compared with the levels without Pap12-6. All experiments were performed three times independently. The error bars represent means ± SEM. (*p < 0.05; **p < 0.01; ***p < 0.001; n.s. represents no significance).

To further investigate the anti-inflammatory activity of Pap12 peptides, we indirectly measured the peptide-induced inhibition of NO production in LPS-stimulated RAW 264.7 macrophages. As shown in Fig. 6A, Pap12-6 most efficiently inhibited NO production (100% inhibition was seen with 20 μM Pap12-6) in LPS-stimulated RAW 264.7 macrophages.

To check the anti-inflammatory activities of the peptides, we measured their abilities to inhibit proinflammatory cytokine production in LPS-stimulated RAW 264.7 cells by enzyme-linked immunosorbent assays (ELISA). Cells were treated with the peptides at 10 and 20 µM, and TNF-α and IL-6 levels were measured and compared with those in the controls. Among all peptides, Pap12-6 showed most significant inhibition of TNF-α, by 74.3% and 83.9% at 10 and 20 μM peptides, respectively (Fig. 6B). Pap12-6 also inhibited IL-6 production by 39.7% and 59.6% at 10 and 20 μM, respectively (Fig. 6C), implying that Pap12-6 is a potent anti-inflammatory agent.

### Pap12-6 inhibits TLR4-mediated inflammatory responses

As described above, Pap12-6 exhibited the highest antibacterial as well as anti-inflammatory activities. Accordingly, we selected Pap12-6 for further studies. Since HEK-Blue™ hTLR4 cells stably express TLR4 and contain a secreted alkaline phosphatase reporter gene (SEAP) located downstream from the NF-κB promoter^37^. When HEK-Blue™hTLR4 cells were stimulated with LPS, TLR4-mediated reporter activity was enhanced in HEK-Blue™ hTLR4 cells, providing the SEAP activity at 5, 10, 25, 50, and 100 uM of Pap12-6. Pap12-6 inhibited 31% of SEAP activity at 25 uM and inhibited 95% at 100 uM as shown in Fig. 6D. The results showed that the cells treated with Pap12-6 showed a significant dose-dependent reduction in the LPS-induced SEAP activity. This result indicated that Pap12-6 shows anti-inflammatory activity via a TLR4-mediated NF-κB signaling pathway.

### Pap12-6 prevented *E. coli* K1-induced septic shock in mice

**Figure 7:**
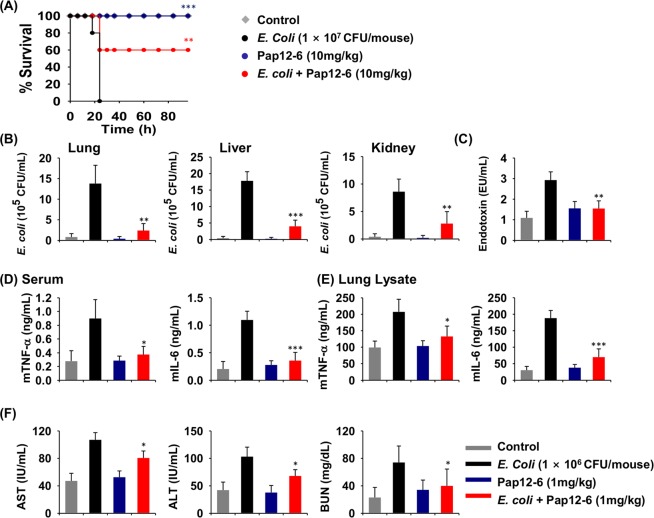
Effects of Pap12-6 on an *E. coli* bacterial sepsis model. (**A**) Survival rates for mice treated with the peptide. To assess the antibacterial effects of Pap12-6, BALB/c mice were intraperitoneally injected with *E. coli* K1 (1 × 10^7^ CFU/mouse) 1 h after 10 mg/kg peptide was intraperitoneally administered. (**B**) Inhibition of bacterial growth in the lung, liver, and kidney of the *E. coli* K1-induced sepsis model. BALB/c mice were pretreated with 1 mg/kg peptide, followed by intraperitoneal injection with *E. coli* (1 × 10^6^ CFU/mouse). (**C**) LPS endotoxin in the serum of the *E. coli* K1-induced sepsis model. (**D**) Inhibition of cytokine production (mTNF-α and mIL-6) in the serum. (**D**) Inhibition of cytokine production (mTNF-α and mIL-6) in lung lysates. (**F**) Serum aspartate aminotransferase (AST), alanine aminotransferase (ALT), and blood urea nitrogen (BUN) levels in the mouse septic shock model with *E. coli*. Four treatment groups in (**B**–**F**) are denoted in grey (PBS), black (1 × 10^6^ CFU/mouse *E. coli*,), blue (1 mg/kg Pap12-6), and red (1 mg/kg Pap12-6 with *E. coli* K1). The error bars represent means ± SEM. (*p < 0.05; **p < 0.01; ***p < 0.001; n.s. represents no significance).

We next investigated the ability of Pap12-6 to prevent Gram-negative bacterial infection by examining its effects on a *E. coli* K1-induced septic shock mouse model. When we administered PBS (control) or 10 mg/kg Pap12-6 to non-infected mice, all mice survived over 4 days, indicating a lack of acute toxicity (Fig. 7A). The survival rate for *E. coli*-infected mice was 0% after 18 h. However, pretreatment with 10 mg/kg Pap12-6 increased the survival rate to 100% at 24 h and to 60% at 96 h post-infection.

Further, we tested the ability of the Pap12-6 peptide to inhibit bacterial growth in the organs of *E. coli* K1-induced septic shock model mice. When we administered 1 mg/kg Pap12-6 1 h before *E. coli* K1 infection, the bacterial counts were significantly reduced in the lung, liver, and kidney of mice by over 82.6%, 77.5%, and 67.4%, respectively, (Fig. 7B), indicating the strong ability of Pap12-6 to inhibit *E. coli* growth *in vivo*. To determine the level of endotoxin in mouse serum, we utilized the LAL assay and found that pretreatment with Pap12-6 significantly reduced the amount of endotoxin by over 47.2% (Fig. 7C). Although Pap12-6 decreased the level of LPS in the serum, additional experiments are needed to clarify the mechanism underlying this neutralising effect.

Next, we tested the ability of the Pap12-6 peptide to suppress the production of pro-inflammatory cytokines in an *E. coli* K1-induced septic shock mouse model. *E. coli* K1 induced significant increases in TNF-α and IL-6 in serum and lung lysates of untreated mice, while Pap12-6 pretreatment was suppressed these *E. coli*-stimulated increases by 58.3% and 67.4%, respectively, in the serum (Fig. 7D) and by 35.9% and 62.9%, respectively, in lung lysates (Fig. 7E).

Further, *E. coli* K1-injected mice were treated with Pap12-6 and the serum levels of aminotransferase (AST), alanine aminotransferase (ALT), and blood urea nitrogen (BUN) were measured. Pap12-6 significantly reduced the *E. coli* K1-induced increases in AST, ALT, and BUN in mice, proving that Pap12-6 can reduce liver and kidney cell damage induced by *E. coli* infection. As shown in Fig. 7F, Pap12-6 reduced the AST, ALT, and BUN levels by 24.7%, 24.4%, and 45.9%, respectively. Taken together, our results show the efficacy of Pap12-6 for the prevention of sepsis.

## Discussion and Conclusion

MDR Gram-negative bacteria have become a major challenge for the development of new antibiotics with broad-spectrum activity for the treatment of sepsis^2,3,7^. AMPs are a promising new class of antibiotics, as they are highly effective against drug-resistant bacteria^38^. Found in a wide range of organisms, they play a crucial role in the innate immune response of higher organisms. Most AMPs kill bacteria by disrupting membrane integrity. Despite the bactericidal effect of AMPs, low cell selectivity and lower biostability are obstacles to their therapeutic applications. Alterations of physiochemical properties, such as the net charge, α-helicity, hydrophobicity, and hydrophobic moment (μ), by changing the sequence and reducing the length of peptides are the most common optimisation techniques for the rational design of potent AMPs^29,39^.

In the current study, we designed new 12-meric short peptides with potency against MDR Gram-negative bacteria. These peptides were derived from N-terminal 12 residues of papiliocin (Pap12-1) and displayed improved antibacterial activity and anti-inflammatory activity compared with those of the parent peptide. Since previous studies have shown that Trp^2^ and Phe^5^ in the amphipathic N-terminal helix of papiliocin are essential for the rapid permeabilization of the Gram-negative bacterial membrane, these residues were retained^23,24^. The net positive charge and amphipathicity of the antimicrobial peptides enable strong electrostatic interactions with the negatively charged outer membrane (mainly LPS) and the phospholipid inner membranes of Gram-negative bacteria. Since all Pap12 analogues have a net charge of +6, which is more positive than Pap12-1 (+4), the enhanced electrostatic interactions with bacterial membranes improved the antibacterial activities of all analogues compared to that of Pap12-1. Given that the mean amphipathic moment (Table 1) was highest for all Pap12-6 peptides, Pap12-6 could destabilise and permeabilize the bacterial membrane and showed more potent antibacterial activities against MDR Gram-negative bacteria, comparable to that of melittin. However, unlike melittin, Pap12-6 exhibited very low cytotoxicity against mammalian cells. Pap12-6 showed the highest TI, which was a three-fold higher than that of Pap12-1 against Gram-negative bacteria.

The adoption of the α-helical structure in membrane-mimicking conditions, as shown in CD spectra (Fig. 3A–C), suggests that Pap12-6 has the tightest interaction with the membrane among all peptides. The indole ring in the Trp side chain tends to partition near the membrane–water interface when inserted into a biological membrane^31–33^. Thus, the additional indole ring in Trp^12^, which facilitates Pap12-6 folding into an amphipathic α-helical structure in the bacterial membrane, likely contributed to its high antimicrobial efficacy.

The mechanism underlying the antibacterial activity of Pap12 peptides was further supported by dye leakage and membrane depolarization experiments as well as electron microscopy. The dye leakage data suggest that Pap12-6 shows relatively strong interactions with bacterial membrane-mimicking LUVs and weak interactions with eukaryotic membrane liposomes. Even though the sequences of Pap12-3 and Pap12-5 differ from that of Pap12-6 only at the 12^th^ position, Pap12-3 and Pap12-5 could not induce the membrane depolarization of *E. coli*. Therefore, compared to Gly and Ala, Trp^12^ at the C-terminus is considerably more efficient in partitioning into the membrane, resulting in effective permeabilization of the *E. coli* cell membrane. These data, together with SEM results for the membrane disruption of *E. coli* by Pap12-6, suggest that Pap12-6 can selectively act on and disrupt bacterial membranes, likely due to the high amphipathicity and Trp at the C-terminus. Further studies are necessary to investigate the antibacterial mechanism of other Pap12 peptides, since they do not effectively depolarize the bacterial membrane. They may penetrate the bacterial cell membrane and target intracellular components.

In cells stimulated by LPS, TLR4 associates with myeloid differentiation protein 2 (MD-2) and forms the TLR4-MD2-LPS complex^40^. TLR4-MD2-LPS formation activates the myeloid differentiation factor 88 (MyD88), leading to the activation of inflammatory proteins, including MAPKs and nuclear factor-κB (NF-κB), which control the transcription of various pro-inflammatory cytokines involved in sepsis^6,40^. Excessive production of pro-inflammatory cytokines causes tissue and organ damage^41^. Pap12-6 also strongly inhibited proinflammatory cytokine production in LPS-stimulated macrophage cells. SEAP activity was inhibited significantly by Pap12-6, implying that Pap12-6 inhibited NF-κB activation and confirming its role in the innate defence response involving TLR4-mediated signaling pathways.

We further evaluated the antibacterial and immunomodulatory effects of Pap12-6 in an *E. coli* K1-induced septic mouse model. Survival of patients with sepsis is heavily dependent on the clearing of bacteria. Pretreatment with Pap12-6 during bacterial infection in mice resulted in the significant inhibition of bacterial growth in the lung, liver, and kidney. Pap12-6 treatment also reduced the level of LPS and the production of TNF-α and IL-6 in the serum and lung lysate in the *E. coli* K1-induced mouse septic shock model. These results suggest that amphipathic Pap12-6, with two tryptophan residues, effectively interacts with negatively charged LPS in the outer membrane of *E. coli* and neutralises pathogenic LPS in the septic shock mouse model, resulting in high antibacterial and anti-inflammatory activities. Thus, Pap12-6 could ameliorate sepsis conditions via bactericidal as well as immunomodulatory effects in the host. This protection, combined with its ability to kill MDR bacteria, makes Pap12-6 an attractive candidate for the treatment of sepsis. Further detailed analyses of the mechanism underlying the anti-inflammatory activities of Pap12-6 are needed. Further experiments with pre-, co- and after-treatments of papiliocin need to be done to prove the potency of Pap12-6 in sepsis, too.

Since sepsis is a systemic multifaceted immune response to infection, which can lead to multiple organ failure and even death^6,7^, there is an urgent need to develop potent anti-sepsis agents with low cytotoxicity. Sepsis-induced mortality and morbidity can be lowered by reducing liver injury and restoring liver function^41^. The liver has a vital role in clearing the infection and mediating the immune response during sepsis. Hypoxic hepatitis, a clinical characteristic of sepsis-associated liver dysfunction, is marked by rapid elevations of AST and ALT^42^. Pap12-6 significantly ameliorated the *E. coli* K1-induced AST, ALT, and BUN serum levels in mice. This ability of Pap12-6 to restore liver and kidney function makes it a promising candidate for sepsis treatment.

The rapidly increasing rate of MDR and sepsis caused by Gram-negative bacteria makes the development of alternative antibiotics an urgent requirement. The novel short, 12-meric Pap12-6 peptide developed in this study permeabilizes the bacterial membrane and is highly effective against MDR Gram-negative bacteria. In addition, Pap12-6 inhibits LPS-stimulated cytokine production in mouse macrophages and likely exhibits anti-inflammatory activity via the TLR4 signalling pathway. It also significantly ameliorates the excessive inflammatory response to Gram-negative bacteria during sepsis, thus preventing organ dysfunction, and significantly inhibits bacterial growth *in vivo*. These results indicate that Pap12-6 could improve the condition of sepsis via dual modes of action, including bactericidal as well as immunomodulatory effects. Therefore, the potency of Pap12-6 against MDR Gram-negative bacteria and its ability to counter Gram-negative sepsis make it an attractive novel peptide for therapeutic applications.

## Methods

### Peptide synthesis

The peptides were synthesized by standard Fmoc-based solid-phase synthesis and purified by RP-HPLC using a C_18_ column as described previously^43^. Final purities of the peptides (>95%) were confirmed using an analytical Vydac C_18_ column (Fig. S1). As shown in Fig. S2, the molecular masses of the peptides were determined by matrix-assisted laser-desorption ionization-time-of-flight (MALDI-TOF) mass spectrometry at the Korea Basic Science Institute (KBSI, Ochang, Korea).

### Bacterial strains

*Escherichia coli* (KCTC 1682), *Pseudomonas aeruginosa* (KCTC 2004), *Acinetobacter baumannii* (KCTC 2508), *Salmonella typhimurium* (KCTC 1926), *Bacillus subtilis* (KCTC 3068), *Staphylococcus aureus* (KCTC 1621), and *Staphylococcus epidermidis* (KCTC 1917) were purchased from the Korean Collection for Type Cultures, Korea Research Institute of Bioscience & Biotechnology (Taejon, Korea). Clinical isolates of MDR *E. coli* (MDREC; CCARM 1229), *P. aeruginosa* (MDRPA; CCARM 2002), *A. baumannii* (MDRAB; CCARM 12035), *S. typhimurium* (MDRST; CCARM 8007), and *S. aureus* (MDRSA; CCARM 3126) were supplied by the Culture Collection of Antibiotic-Resistant Microbe (CCARM) at Seoul Women’s University in Korea. The *E. coli* K1 strain RS218 (O18:K1:H7) was kindly provided by Dr. Jang-Won Yoon of Kangwon National University (Gangwon-do, Korea).

### Antimicrobial activity *in vitro*

Minimum inhibitory concentration (MIC) assays using peptone broth dilution were used to determine the concentration that completely inhibited bacterial growth, as reported previously^23^. Peptides were treated with 2 × 10^6^ CFU/mL bacterial suspensions in 1% peptone media for 16 h at 37 °C.

### Cytotoxicity

The toxicity of peptides to human red blood cells (hRBCs) was determined by measuring the peptide-induced release of haem, and percent hemolysis was calculated as described previously^23^. The peptide-induced cytotoxicity of mouse RAW 264.7 macrophages, human keratinocyte HaCaT cells, and HEK-293 cells were determined by 3-(4,5-dimethylthiazol-2-yl)-2,5-diphenyltetrazolium bromide (MTT) assays as previously described^23^.

### Circular dichroism analysis of peptide secondary structures

The secondary structures of Pap12 peptides were studied by circular dichroism (CD) spectroscopy. A J810 spectropolarimeter (Jasco, Tokyo, Japan) with a 1-mm path length cell was used to record the spectra of peptides (50 μM) in water, 50 mM dodecylphosphocholine (DPC) micelles, and 100 mM sodium dodecyl sulphate (SDS) micelles at 25 °C from 190 to 250 nm at 0.1-nm intervals. Each spectrum was obtained by averaging 3 scans and further smoothed using J810 software. CD data are shown as the mean residue ellipticity (θ) in degrees·cm^2^·dmol^−1^.

The secondary structures of the peptides were modelled using the *de novo* peptide structure prediction server “PEP-FOLD” in the RPBS portal (http://mobyle.rpbs.univ-paris-diderot.fr/cgi-bin/portal.py#forms::PEP-FOLD, Fig. S3)^34–36^. Using amino acid sequences as input data, the best 5 clusters from 100 simulations were obtained for each peptide and the lowest energy conformations were selected.

### Calcein dye leakage assay

To check the ability of the peptides to permeabilize the membranes, bacterial and mammalian membrane models were prepared. Large unilamellar vesicles (LUVs) composed of egg yolk l-α-phosphatidylcholine (PC)/egg yolk l-α-phosphatidyl-dl-glycerol (PG) (7:3, w/w) with calcein dye were prepared as described previously^43^. Mammalian membrane-mimicking LUVs were prepared using PC/cholesterol (CH) (10:1, w/w). Calcein release from LUVs upon the addition of different concentrations of peptides was measured using a RF-5301PC spectrofluorophotometer (Shimadzu, Kyoto, Japan) with an excitation wavelength of 490 nm and an emission wavelength of 520 nm. Complete calcein release was obtained by 1% Triton X-100 (10 μL) and the percentage was calculated as described previously^43^.

### Membrane depolarization

Peptide-induced membrane depolarization in *E. coli* cells was measured as described previously^44^. The membrane potential changes were measured using 3,3′-dipropylthiadicarbocyanine iodide (DiSC_3_(5)). Triton X-100 (1 µL; 1%) was used to measure the complete disruption of membrane potential and membrane depolarization was calculated as described previously^44^.

Peptide-induced inner membrane permeability was measured using *o*-nitrophenyl-β-d-galactopyranoside (ONPG). Freshly grown *E. coli* ML35p was harvested at mid log phase and washed two times in 10 mM sodium phosphate buffer with 100 mM NaCl at pH 7.4 and suspended in the same buffer containing 1.5 mM ONPG. This bacterial solution (100 µL) was added to serially diluted peptides (100 µL), prepared in the same buffer as the bacterial suspension, and further incubated for 20 min at 25 °C. Absorbance was read at 420 nm and wells with or without bacteria were used as the controls.

### SEM analysis

*E. coli* ATCC25922 was cultured in Mueller Hinton broth at 37 °C until reaching the mid-log phase. After centrifugation, the cells were washed and diluted in 10 mM PBS to an OD_600_ of 0.2. The cells were incubated with 1× MIC or 2× MIC of Pap12-6 for 2 h. After 2 h, the cells were washed three times using 10 mM PBS and fixed in 2.5% glutaraldehyde overnight at 4 °C. The cells were washed and fixed in 1% osmium tetroxide for 1 h. The fixed cells were dehydrated by a series of graded ethanol and were moved to mixture of ethanol and isoamyl acetate and then pure isoamyl acetate for 10 min at each step. Finally, the cells were dehydrated, coated with gold, and visualised by SEM (Super 55vp; Carl Zeiss, Oberkochen, Germany) at KBSI, Chucheon.

### Quantification of nitrite and inflammatory cytokine production in LPS-stimulated RAW 264.7 cells

Nitrite accumulation in the culture medium was used as an indicator of NO production. RAW 264.7 cells were plated at a density of 1 × 10^5^ cells/well and stimulated with 50 ng/mL LPS from *E. coli* O111:B4 (Sigma-Aldrich, St. Louis, MO, USA) in the presence or absence of peptides for 16 h. Nitrite production was determined by measuring absorbance at 540 nm and was converted to the nitrite concentration by reference to a standard curve generated with NaNO_2_^45^.

The peptide-mediated inhibition of the expression of tumor necrosis factor-α (TNF-α) and Interleukin-6 (IL-6) in LPS-stimulated RAW 264.7 cells was determined by enzyme-linked immunosorbent assays (ELISA; R&D Systems, Minneapolis, MN, USA) as described previously^45^.

### Effect of papiliocin on TLR4-mediated SEAP activity in LPS-stimulated HEK-Blue™ hTLR4 cells

HEK-Blue™ hTLR4 cells (IvivoGen, San Diego, CA, USA) were seeded in 96-well plates at a density of 2.5 × 10^4^ cells/well with Pap12-6 in HEK-Blue detection media (IvivoGen, San Diego, CA, USA). After 1 hr, cells were treated by LPS (20 mg/ml). After 14 h, SEAP activity was determined by measuring absorbance at 630 nm with the ELISA reader.

### Sepsis mouse model

Female BALB/c (6 weeks old) mice were used for the *E. coli* K1-induced sepsis model. All mice were obtained from Orient (Daejeon, Korea) and were housed under specific pathogen-free (SPF) conditions in a humidity- and temperature-controlled environment for 1 week prior to the experiments. All procedures were reviewed and approved by the Institutional Animal Care and Use Committee (IACUC) of Konkuk University, South Korea (IACUC number: KU17044).

### Survival test using the sepsis mouse model

To determine the *in vivo* survival rate, four groups were established with 10 BALB/c mice per group. For the control group, 10 mice were given an intraperitoneal (i.p.) injection with PBS. In the second group, 10 mice were given an i.p. injection with 0.2 mL of *E. coli* K1 (1 × 10^7^ CFU/mouse). In the third group, 10 mice were i.p. injected with Pap12-6 (10 mg/kg). In the fourth group, 10 mice were i.p. injected with Pap12-6 (10 mg/kg) 1 h before the i.p. injection of *E. coli* K1 (1 × 10^7^ CFU/mouse). Survival was evaluated for 4 days (0, 6, 12, 18, 24, 30, 36, 48, 60, 72, 84, and 96 h). During the *in-vitro* and *in-vivo* experiments, all peptides were stable and retained their activities, providing evidence for their stability over these periods. Further pharmacokinetic analyses need to be done to confirm peptide stability.

### Cytokine levels in the serum or lung lysate in the sepsis mouse model

BALB/c mice (5 mice per group) were i.p. injected with the peptide (1 mg/kg) 1 h before the i.p. injection of *E. coli* K1 (1 × 10^6^ CFU/mouse). Quantities of TNF-α and IL-6 in the mouse serum were determined using sandwich ELISA kits. Three control groups consisting of mice without any treatment, treated only with peptides, and treated only with *E. coli* were used and the experiments were performed three times independently.

### Determination of bacterial counts in organ tissues

At the time of sacrifice, the lungs, liver, and kidney were removed aseptically and placed separately in 1 mL of sterile PBS. Tissues were then homogenised under a vented hood on ice and homogenates of the lung, liver, and kidney were diluted with PBS at 1:1000. After adding 10 µL of each diluted sample to an LB agar plate, colonies after 1 day of incubation at 37 °C were counted.

### Detection of AST, ALT, and BUN levels in mouse serum

Aspartate aminotransferase (AST), alanine amino transferase (ALT), and blood urea nitrogen (BUN) in the serum were measured using standard kits available from Asan Pharmaceutical (Seoul, Korea) following previously described methods^46^.

### LAL assay of LPS endotoxins in mouse serum

Endotoxin levels in mouse serum were measured by an LAL chromogenic endpoint assay (Lonza Group Ltd., Allendale, NJ, USA) according to the manufacturer’s instructions. After subtracting background levels, LPS in EU/mL (endotoxin unit/mL) was determined relative to an endotoxin standard for *E. coli* provided in the assay kit. Absorbance was measured at 405 nm. PBS was used as the negative control and LPS (10 ng/µL) was used as the positive control to calculate the rates.

### Statistical analyses

All measurements were obtained at least three times and statistical analyses were performed using GraphPad Prism (GraphPad Software Inc., La Jolla, CA, USA). All data are presented as means ± S.E.M. from independent experiments. For all statistical analyses except survival test, one-way ANOVA followed by post hoc Bonferroni tests (Prism 7.0) were used to compare treatment groups. For *in-vivo* survival test, Kaplan−Meier survival curves were analyzed using Log-rank tests. Differences were considered statistically significant at p < 0.05.

### Declaration

The experiments and methods associated with human blood were carried out in accordance with the relevant guidelines. Protocols for measurement were approved by the Institutional Review Board for Research Involving Human Research Subjects, Konkuk University. All experimental protocols were approved by the ethics committee of Konkuk University. All animal experiment procedures were reviewed and approved by the Institutional Animal Care and Use Committee (IACUC) of Konkuk University. All methods for the animal study were performed in accordance with the relevant guidelines and regulations. Informed consent was obtained from all subjects.

## Supplementary information

Supplementary Material

## Data Availability

All data generated or analysed during this study are included in this published article. The datasets generated during and/or analysed during the study are available from the corresponding author on reasonable request.
