# Supplementary material for: Development of a novel short 12-meric papiliocin-derived peptide that is effective against Gram-negative sepsis
Source: Sci Rep. 2019 Mar 7;9:3817. doi: 10.1038/s41598-019-40577-8 (PMC6405874; doi:10.1038/s41598-019-40577-8)
Supplement: Supplementary file 1 — Supplementary Material [file 41598_2019_40577_MOESM1_ESM.docx]

**Supplementary Material**

**Development of a novel short 12-meric papiliocin-derived peptide that is effective against gram-negative sepsis**

Jieun Kim ^a,∥^, Binu Jacob^a,∥^, Mihee Jang ^a^, Yeongjoon Lee^a^, Chulhee Kwak ^a^, Kkabi Son ^a^, Sujin Lee ^b^, In Duk Jung ^b^, Myeong Seon Jeong^c^, Seung-Hae Kwon ^c^, and Yangmee Kim^a,^*

^a^Department of Bioscience and Biotechnology, Konkuk University, Seoul 05029, South Korea

^b^Department of Immunology, Lab of Dendritic Cell Differentiation and Regulation, School of Medicine, Konkuk University, Chungju, 27478, South Korea

^c^ Chuncheon Center, Korea Basic Science Institute, Chuncheon 24341, Korea

^*^**Corresponding author: Yangmee Kim, Ph.D (**[**ymkim@konkuk.ac.kr**](mailto:ymkim@konkuk.ac.kr)**)**

Department of Bioscience and Biotechnology, Konkuk University, Seoul 05029, South Korea. <Tel:+822-450-3421>

^∥^ These authors contributed equally to this work.

**Contents**

|  |
| --- |
|  |
| **Figure S1.** HPLC characterisation of all synthetic peptides listed in Table 1.  **Figure S2.** MALDI-TOF characterisation of all peptides listed in Table 1.  **Figure S3.** Lowest energy conformations of the best clusters for the peptides calculated by PEP-FOLD. Hydrophilic residues are indicated in blue(basic) and red(acidic), and hydrophobic residues are shown in yellow. |

Figure S1. HPLC characterisation of all synthetic peptides listed in Table 1.

(A) Pap12-1


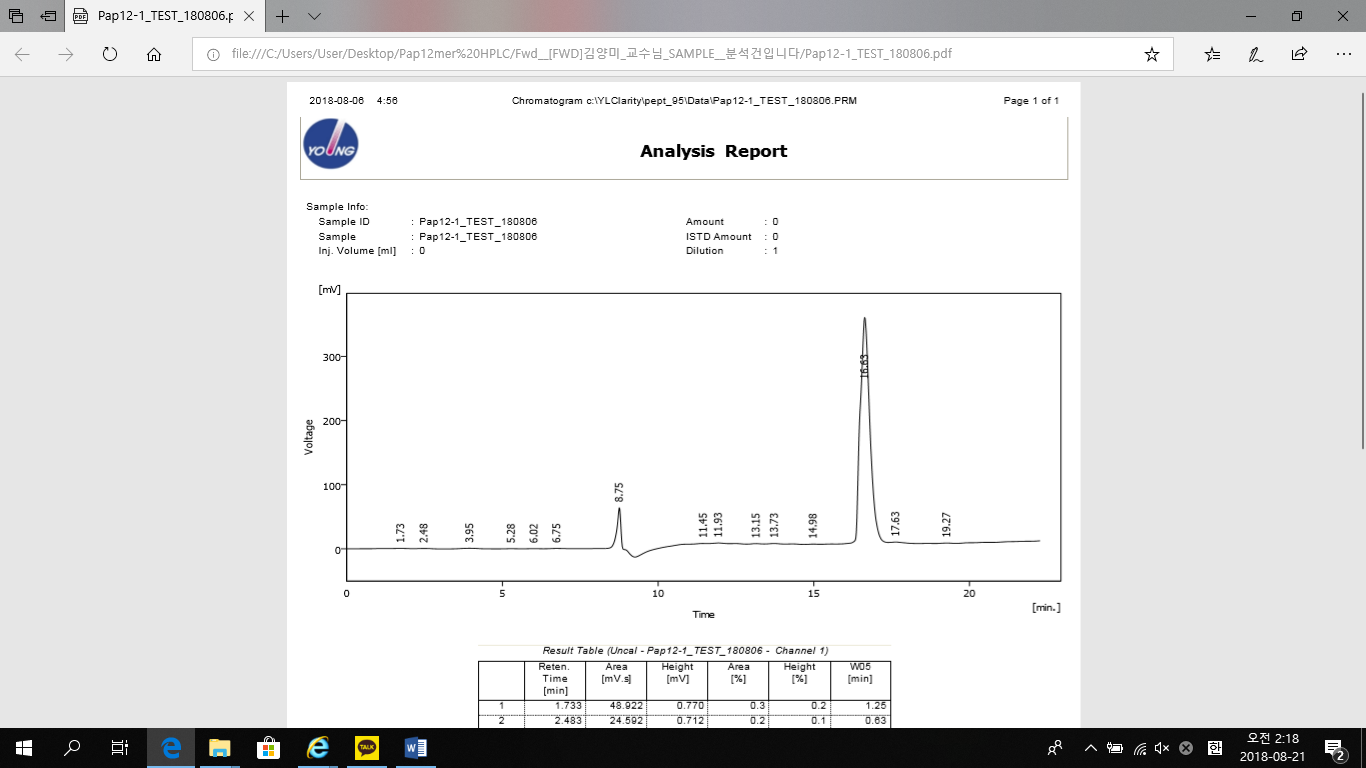


(B) Pap12-2


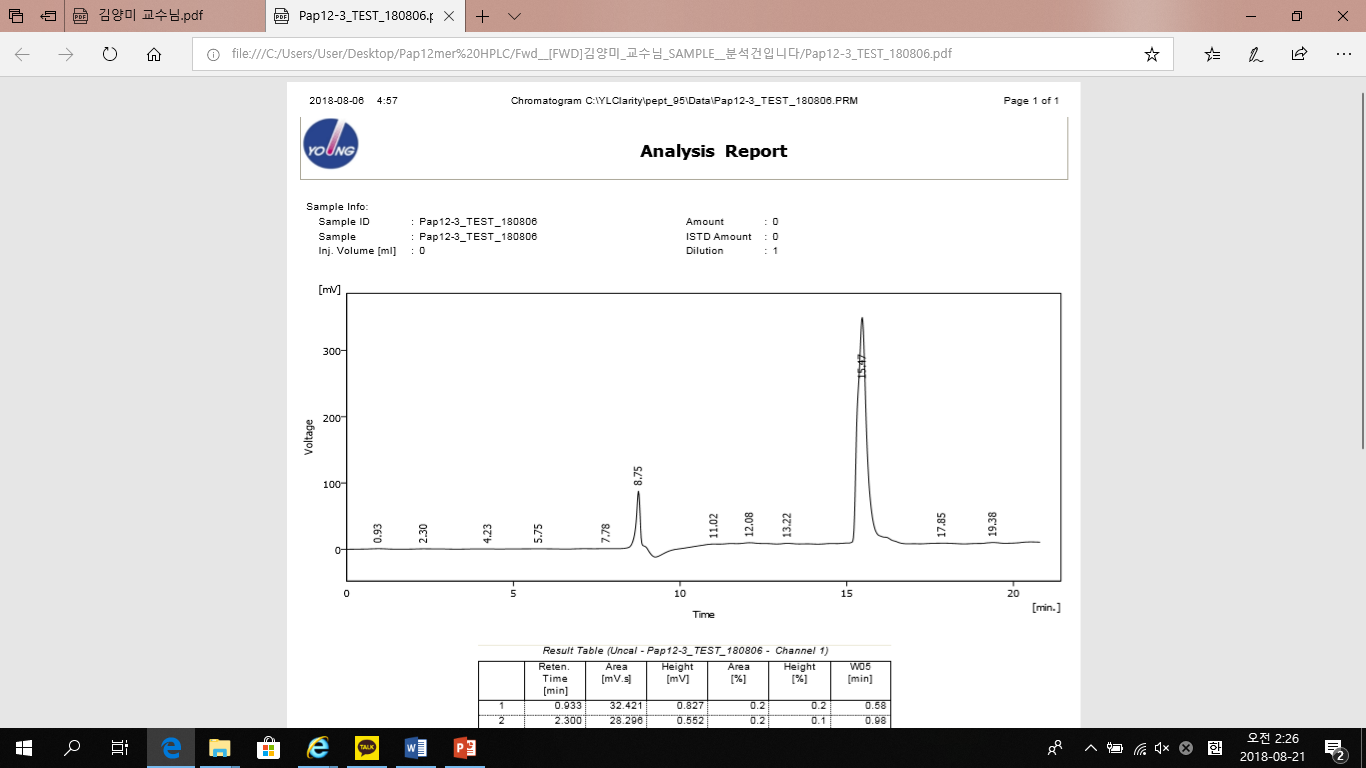


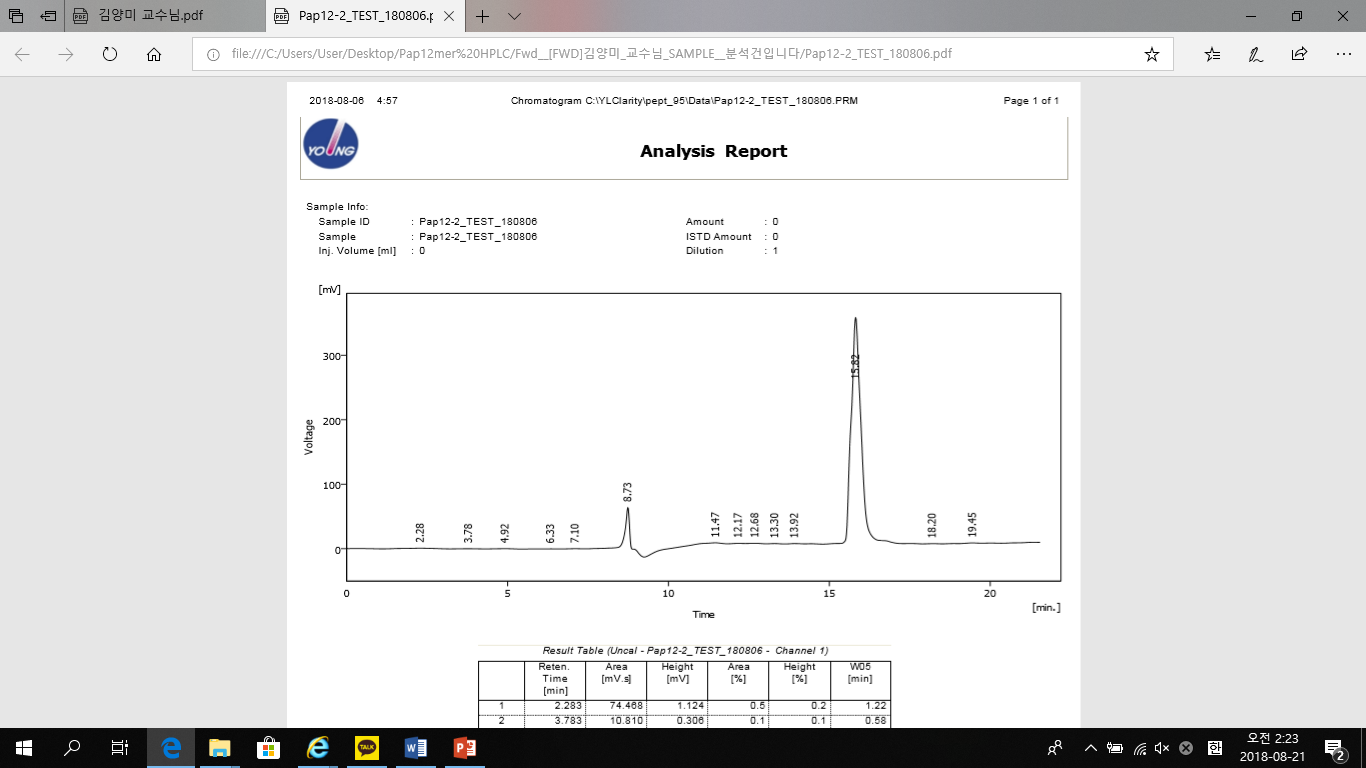


(C) Pap12-3

(D) Pap12-4


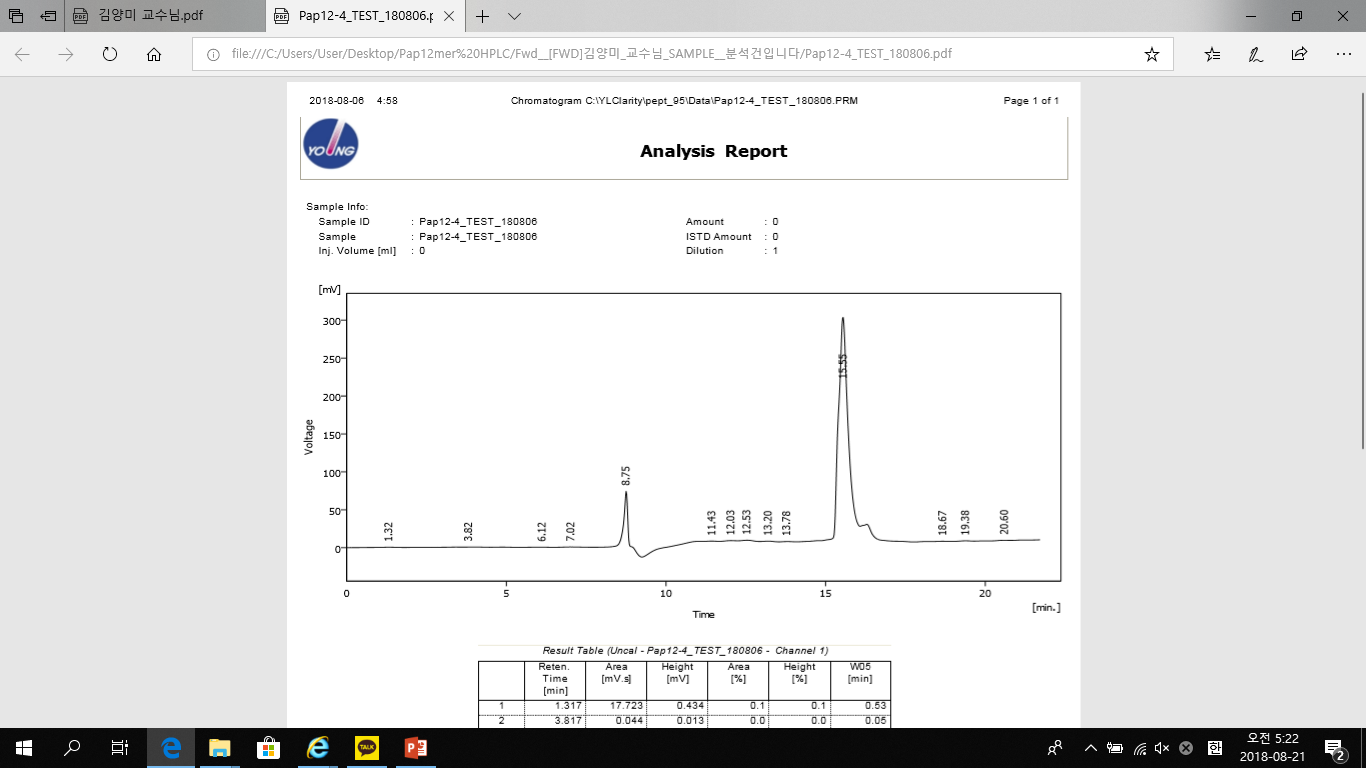


(E) Pap12-5


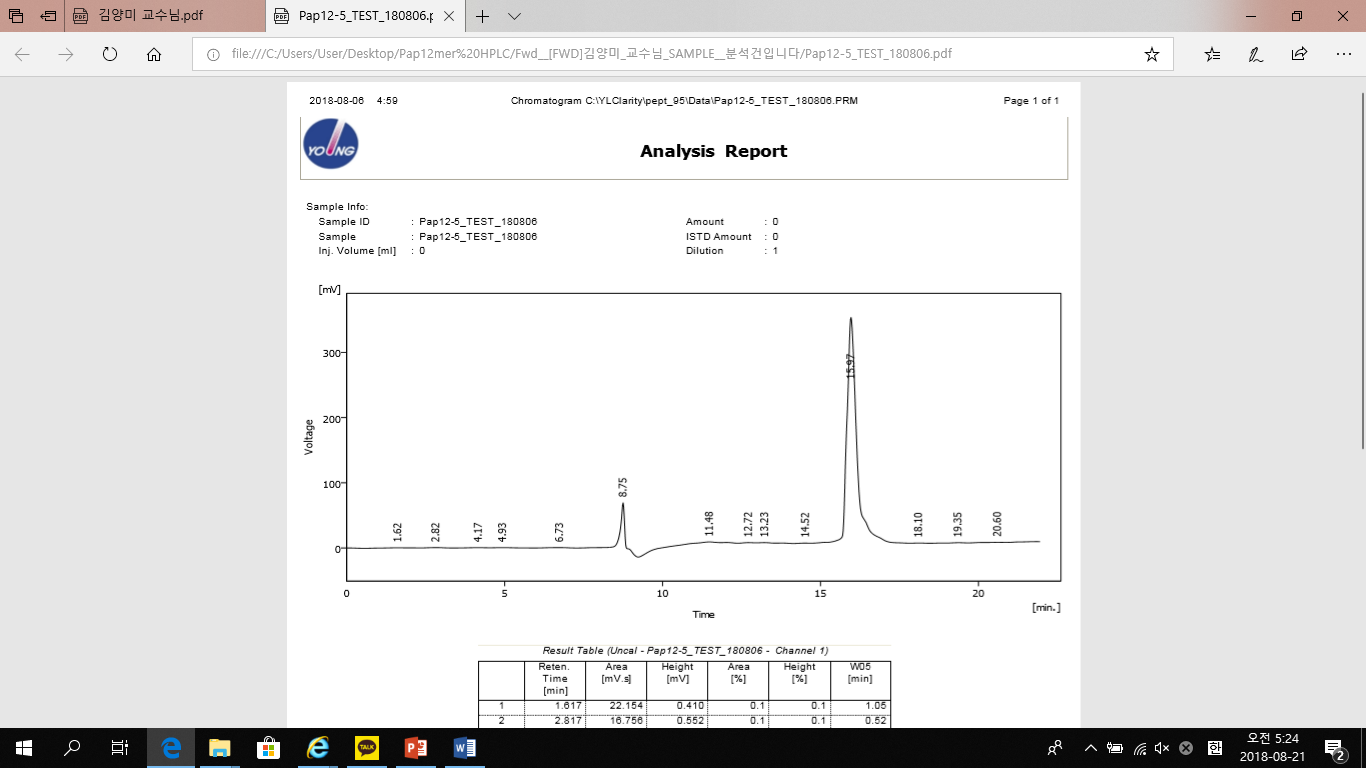


(F) Pap12-6


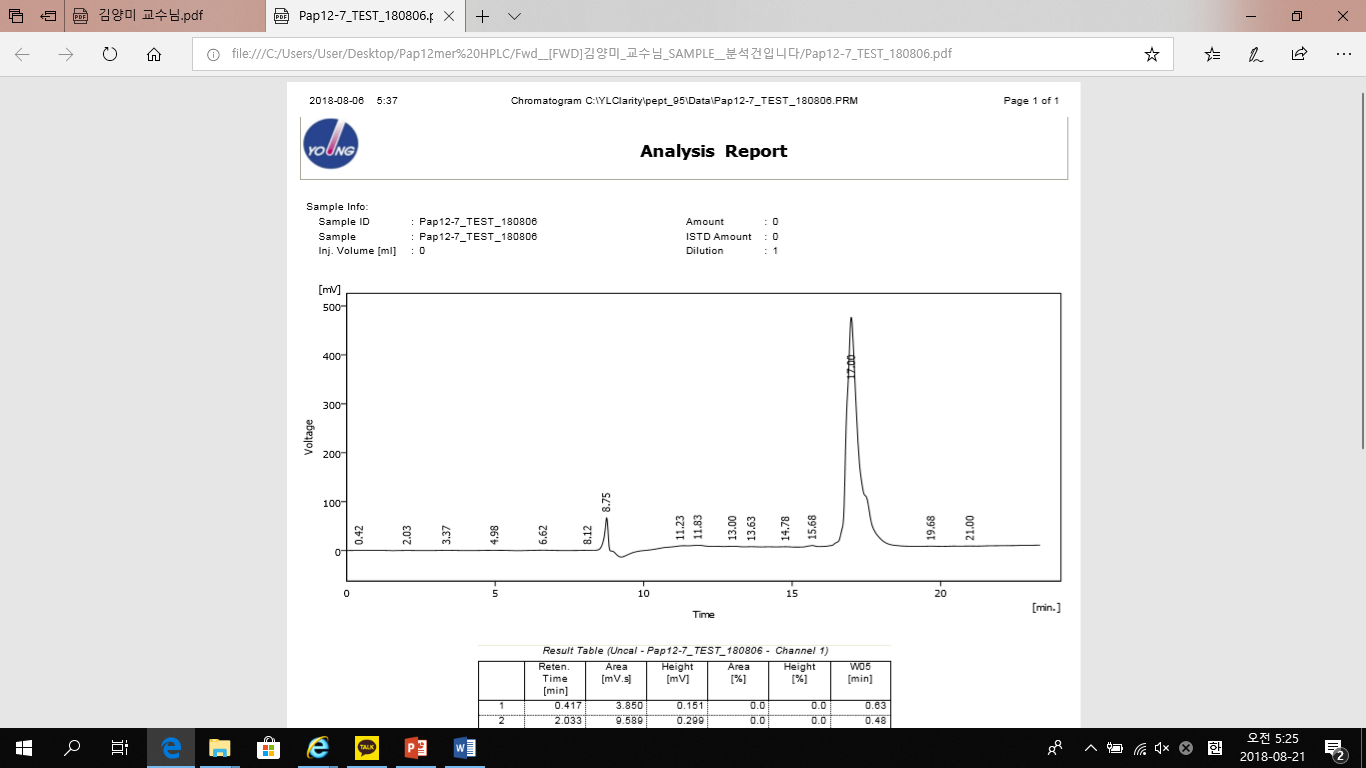


**Figure S2.** MALDI-TOF characterisation of all peptides listed in Table 1.


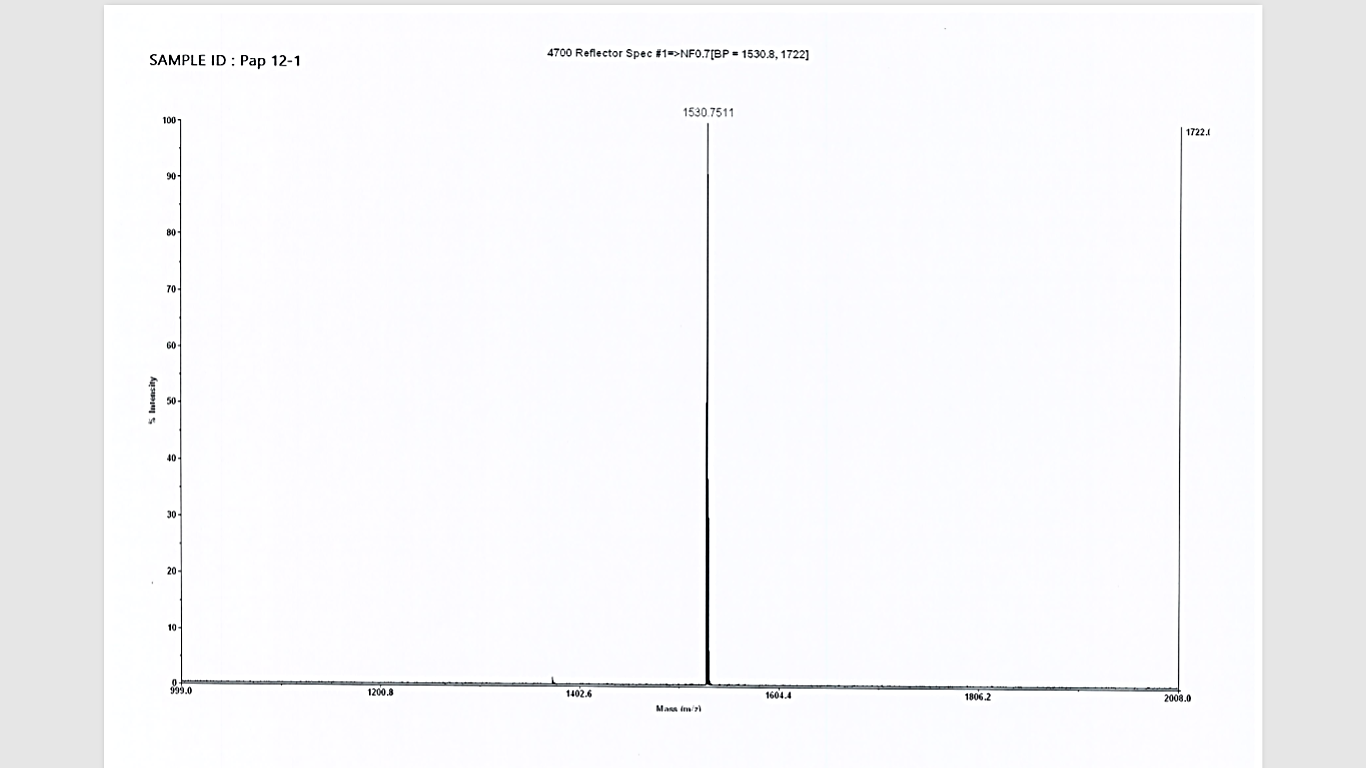


(A) Pap12-1


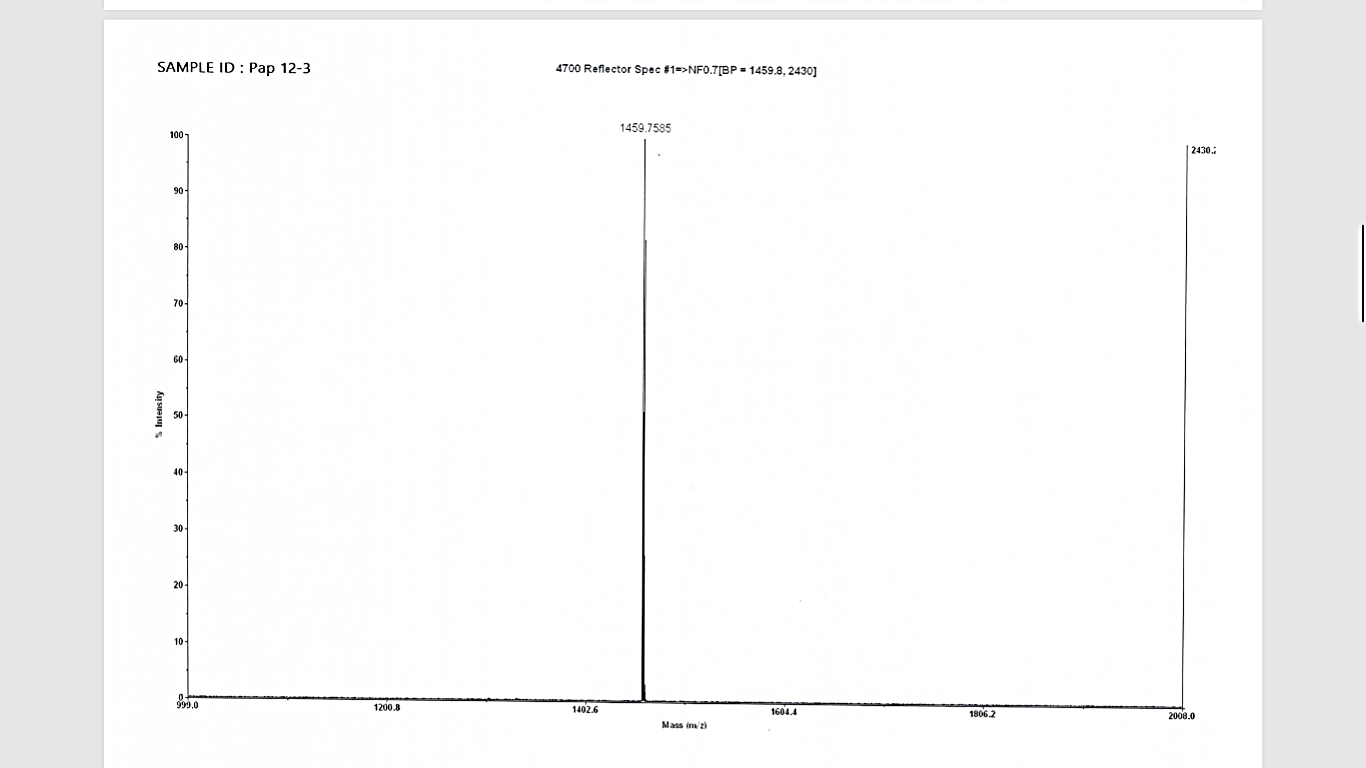


(B) Pap12-2


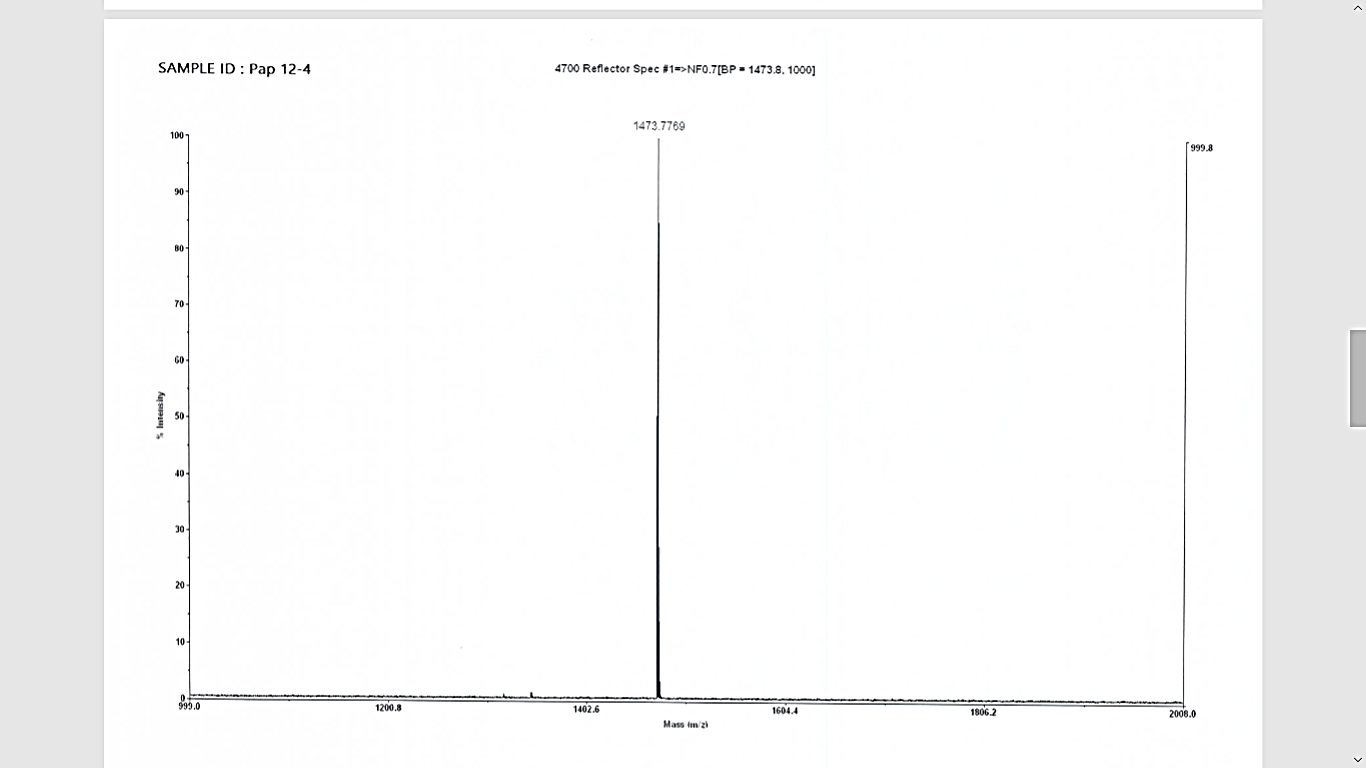

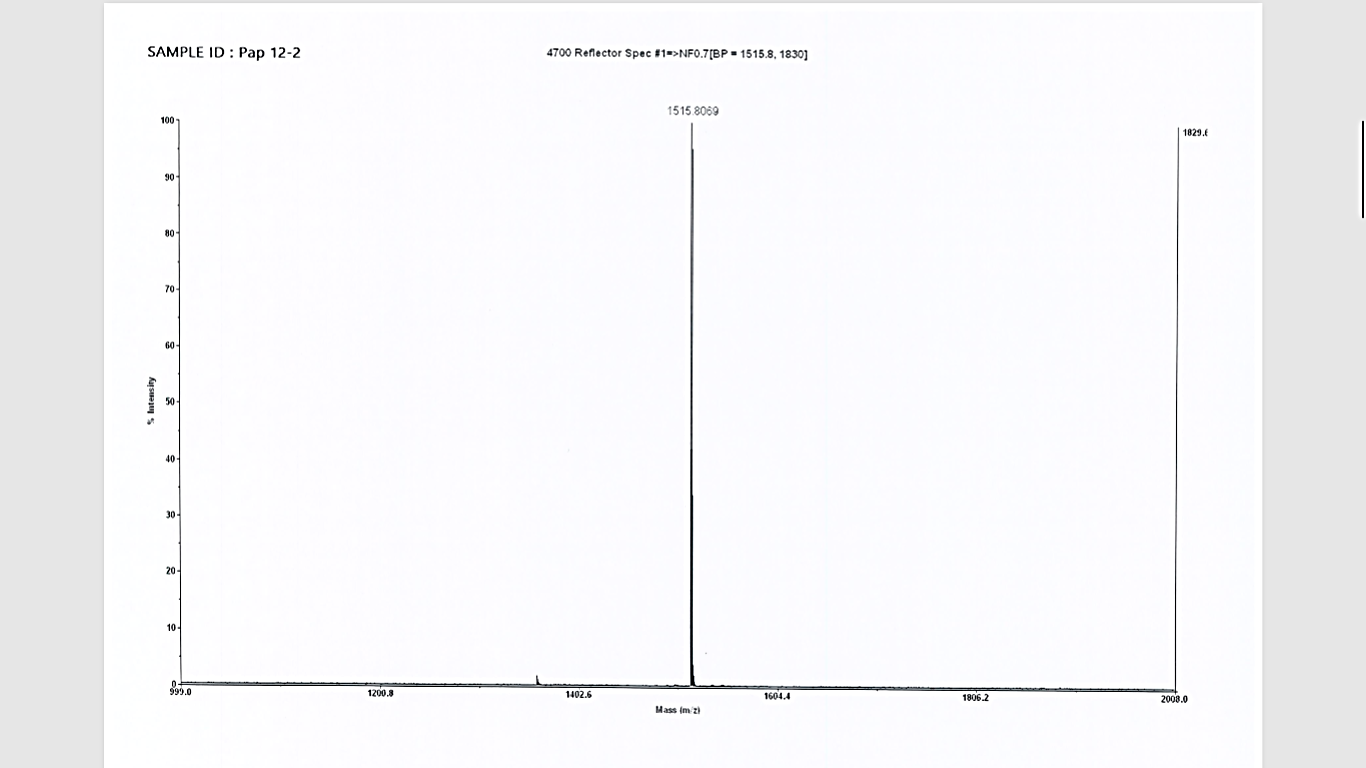


(D) Pap12-4

(C) Pap12-3

(E) Pap12-5


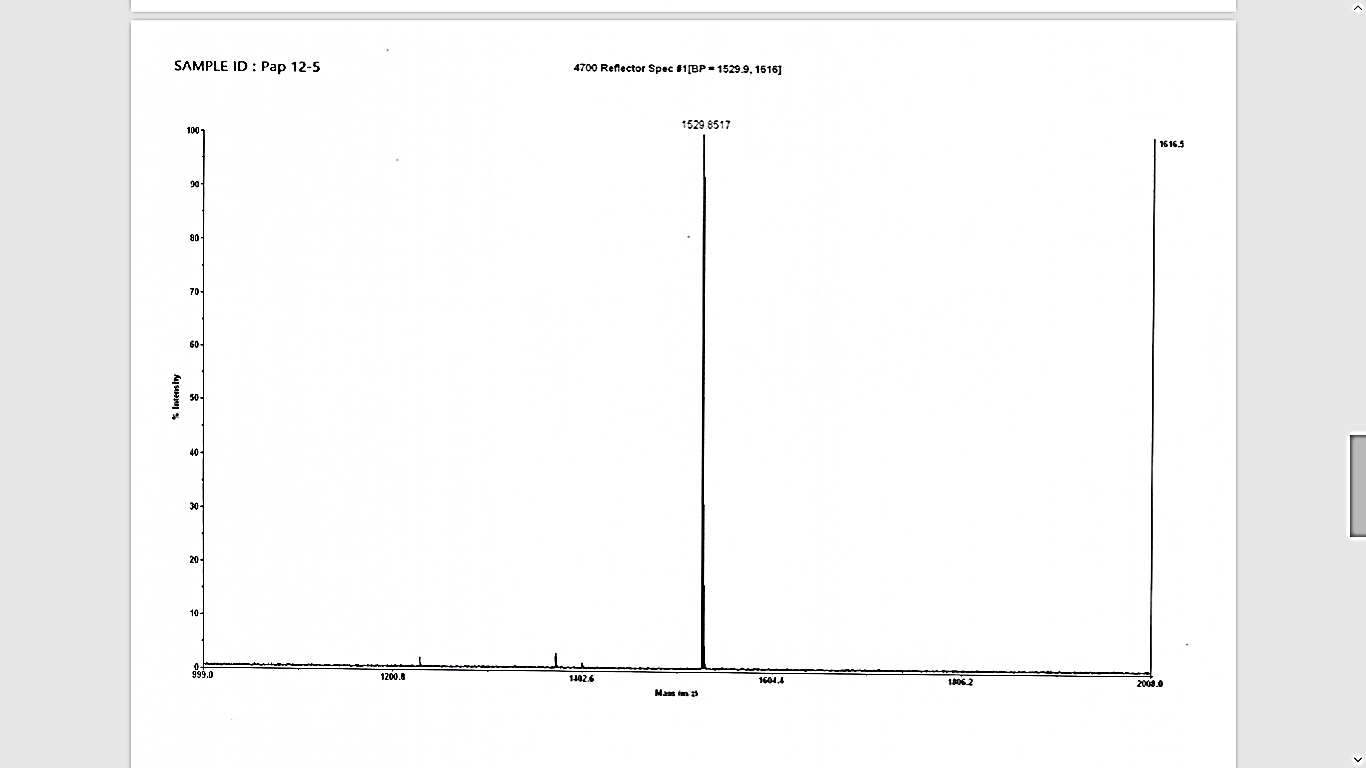


(F) Pap12-6


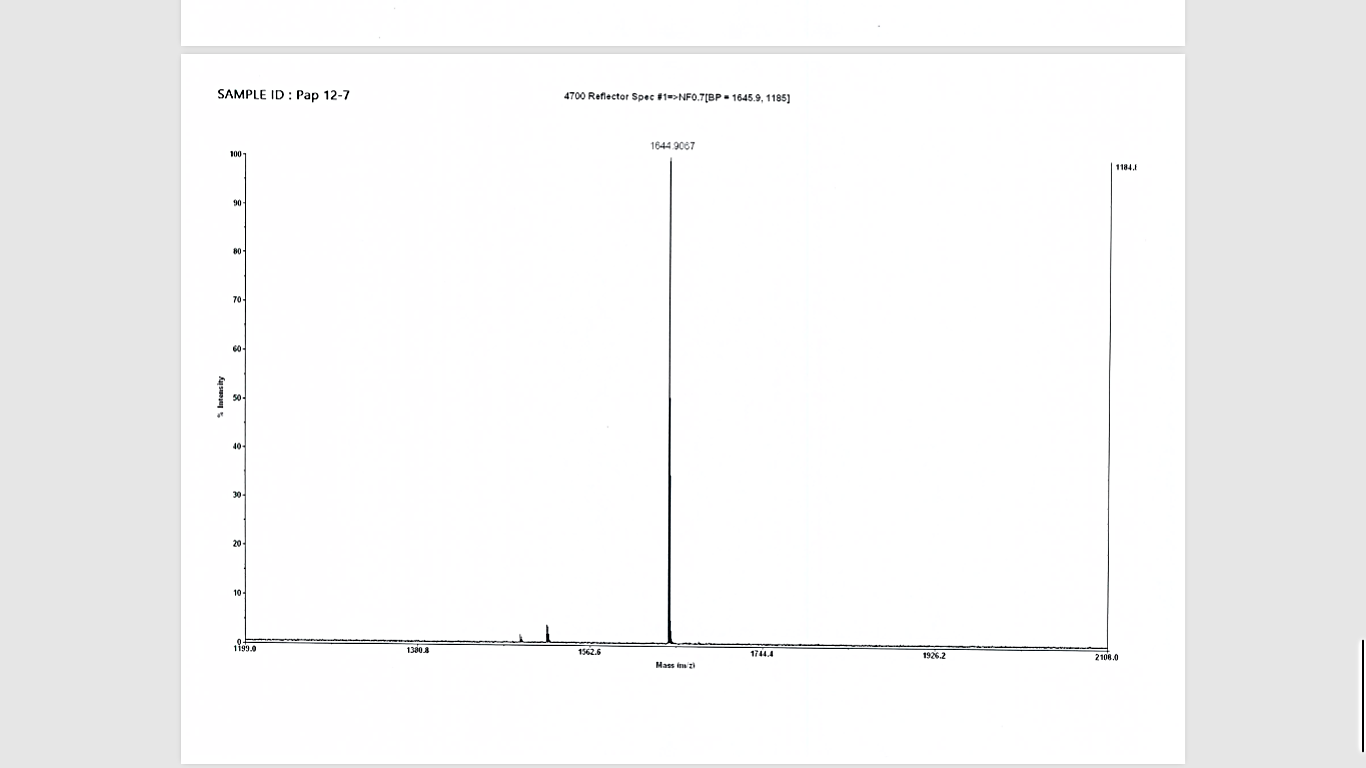


**Figure S3.** Lowest energy conformations of the best clusters for the peptides calculated by PEP-FOLD. Hydrophilic residues are indicated in blue (basic) and red (acidic), and hydrophobic residues are shown in yellow.


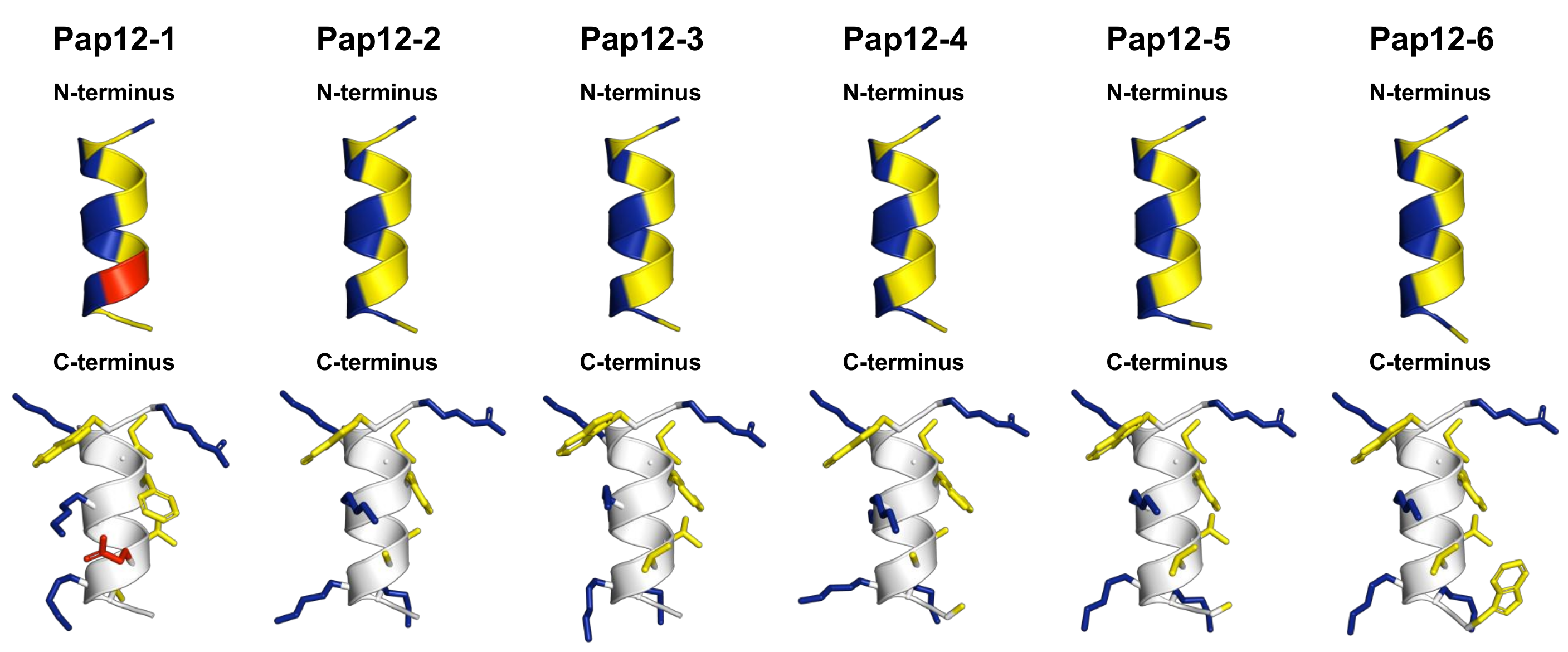


**Results**

As determined by CD experiments, all peptides from Pap12-1 to Pap12-6 exhibited folding as α-helical structures in the presence of lipid micelles. We performed molecular modelling to predict the secondary structures of the peptides using PEP-FOLD, a *de novo* peptide structure prediction server^1-3^. The lowest energy conformations of the best clusters for the peptides are shown in Figure. S3. For all models, the positively charged residues, Arg and Lys, are indicated in blue and the negatively charged Glu is shown in red. The nonpolar residues Trp, Phe, Ile, Val, and Gly are shown in yellow. All peptides had α-helical structures and amphipathic properties.

**Methods**

The secondary structures of the peptides were modelled using the *de novo* peptide structure prediction server PEP-FOLD in RPBS portal (<http://mobyle.rpbs.univ-paris-diderot.fr/cgi-bin/portal.py#forms::PEP-FOLD>).^1-3^ Using amino acid sequences as input data, the best 5 clusters from 100 simulations were obtained for each peptide, and the lowest energy conformations were selected.

1. Néron, B. *et al.* Mobyle: a new full web bioinformatics framework., 3005-3011 (2009).

2. Alland, C. *et al.* RPBS: a web resource for structural bioinformatics. *Nucleic Acids Res*, W44-49 (2005).

3. Thévenet, P. *et al.* PEP-FOLD: an updated de novo structure prediction server for both linear and disulfide bonded cyclic peptides. *Nucleic Acids Res*, W288-293 (2012).
